# Effects of adding ruminal propionate on dry matter intake and glucose metabolism in steers fed a finishing ration

**DOI:** 10.1093/jas/skad072

**Published:** 2023-04-13

**Authors:** Abigail R Rathert-Williams, Hunter L McConnell, Carlee M Salisbury, Amanda K Lindholm-Perry, David L Lalman, Adel Pezeshki, Andrew P Foote

**Affiliations:** Department of Animal and Food Sciences, Oklahoma State University, Stillwater, OK 74078, USA; Department of Animal and Food Sciences, Oklahoma State University, Stillwater, OK 74078, USA; Department of Animal and Food Sciences, Oklahoma State University, Stillwater, OK 74078, USA; USDA, ARS, U. S. Meat Animal Research Center, Clay Center, NE 689332, USA; Department of Animal and Food Sciences, Oklahoma State University, Stillwater, OK 74078, USA; Department of Animal and Food Sciences, Oklahoma State University, Stillwater, OK 74078, USA; Department of Animal and Food Sciences, Oklahoma State University, Stillwater, OK 74078, USA

**Keywords:** cattle, feeding behavior, finishing diet, glucose metabolism, intravenous glucose tolerance test, propionate

## Abstract

The objective of this experiment was to determine if supplying additional propionate to the rumen alters dry matter intake (**DMI**), feeding behavior, glucose metabolism, and rumen fluid metabolites in steers fed a finishing diet. Ruminally cannulated steers (*n* = 6) were fed a finishing diet ad libitum. Steers were randomly assigned to one of three treatments in a 3 × 6 Latin rectangle design with three 15 d periods. Treatments of no Ca propionate (Control), 100 g/d (Low), or 300 g/d (High) were ruminally dosed twice daily. Individual intake was measured using an Insentec feeding system. Pre-feeding blood samples were collected on day 7 and rumen fluid samples were collected on day 13. An intravenous glucose tolerance test (**IVGTT**) was conducted on day 14 and liver biopsies were collected on day 15. Liver samples were analyzed for expression of genes involved in gluconeogenesis. Data were analyzed using a mixed model with period, treatment, day, and their interaction included, with day and minute within period as a repeated measure and steer as a random effect. Meal size (*P* = 0.049), meal frequency (*P* = 0.046), and DMI (*P* < 0.001) were decreased in High steers. Day 7 plasma glucose (*P* = 0.23) and lactate (*P* = 0.47) were not affected by treatment, but insulin was decreased (*P* = 0.008) and non-esterified fatty acids were increased (*P* = 0.044) in the High treatment compared with the Control. Rumen fluid lactate was decreased (*P* = 0.015) in the High treatment compared with the Low treatment. Total VFA concentrations did not differ (*P* = 0.88) between treatments. There was treatment × time interaction for proportions of acetate and propionate (*P* < 0.001) and the acetate:propionate ratio (*P* = 0.005). The effect on acetate was due to a decrease in the High treatment 2 h after dosing the treatment. Propionate proportions were greater in the High treatment than the Control at all time points and differed from the Low except at 0 h. Propionate treatments had no major effects on the glucose and insulin parameters observed in the IVGTT other than a tendency (*P* = 0.09) for an increased insulin time to peak. These data indicate that exogenous propionate decreases DMI but the decrease in propionate from fermentation due to reduced DMI might negate the supply of exogenous propionate in VFA supply to the animal. Mechanisms other than hepatic oxidation of propionate might be responsible for DMI regulation.

## Teaser

Supplying propionate to the rumen of steers results in a decrease in feed intake, but the decrease in feed intake likely negates the exogenous propionate in total propionate supply, indicating that alternative mechanisms might regulate feed intake of finishing beef cattle.

## Introduction

Microbial metabolism of dietary carbohydrates in the rumen limits the availability of glucose to be absorbed. The volatile fatty acids (**VFA**) produced from microbial fermentation are used as precursors for gluconeogenesis in the ruminant liver, with propionate providing 60% to 74% of the carbon for glucose (Aschenbach et al., 2010). Previous research investigating the relationship between increasing propionate supply and dry matter intake (**DMI**) is largely limited to prepartum or lactating dairy cattle on a high forage diet (Oba and Allen, 2003b; DeFrain et al., 2005). It has been shown that diet has a large impact on VFA production, altering the ratio of ­acetate:propionate (Bauman et al., 1971; Wang et al., 2020). On a forage-based diet, acetate is produced in greater proportions than propionate (65:25:10, acetate:propionate:butyrate) and this balance shifts as the inclusion rate of concentrates increases, leading to greater quantities of propionate produced (50:40:10; Owens and Goetsch, 1993). It is unclear if the effects of propionate supply on DMI would be similar in cattle-fed diets with greater amounts of concentrate and therefore producing more propionate from fermentation.

Alterations in DMI are variable when propionate is infused or fed. Depending on the stage of production, propionate either has decreased DMI in late lactation (Oba and Allen, 2003b) or did not change DMI in early lactating cows (DeFrain et al., 2005). In beef heifers fed a grass hay and straw diet, 200 g/d propionate did not influence DMI although less hay was required to achieve similar weight gain as control-fed animals (Lalman et al., 1993). Variations in feed intake caused by increased propionate could be impacted by energy requirements of ruminants at different production stages. Oba and Allen (2003b) reported a decrease in metabolizable energy intake with increasing propionate infusion which negates the idea that ruminants are eating to a certain energy requirement.

The hepatic oxidation theory (**HOT**) describes the role of the ruminant liver in controlling feed intake with hepatic oxidation of non-esterified fatty acids (**NEFA**), lactate, and propionate (Allen, 2014). The effect of propionate as an oxidative fuel in the liver was seen by Anil and Forbes (1988) when denervation of the liver prevented the hypophagic effects of propionate. The rapid metabolism of propionate by the liver leads to the thought that propionate may have a larger impact on meal size, with other oxidative fuels altering meal frequency such as NEFA and lactate (Allen, 2014). Again, as much of this research has utilized lactating dairy cows eating a forage-based diet, little is known about how these factors would alter feeding behavior with a highly fermentable concentrate-based diet.

In addition to DMI varying between studies, the impact of increasing ruminal propionate on the metabolism of the oxidative fuels has been inconsistent. Some have seen no impact of exogenous propionate on plasma NEFA or glucose (DeFrain et al., 2005; McNamara and Valdez, 2005; Ferreira and Bittar, 2011). An increase in plasma NEFA is often seen in early lactation dairy cows due to the dramatic increase in energy requirements with a decrease or no change in DMI (Bell, 1995). Therefore the decrease in plasma NEFA seen by DiCostanzo et al. (1999) would be plausible if propionate is providing the additional energy. Increased plasma glucose and insulin with exogenous ruminal propionate have also been reported (DiCostanzo et al., 1999; Oba and Allen, 2003a; Liu et al., 2010). Feeding propionate to steers consuming a high-concentrate diet was shown to decrease insulin sensitivity by increasing the insulin response to an intravenous glucose tolerance test (**IVGTT**; Rathert-Williams et al., 2021), but there is little additional data on the effect of increasing propionate flux to the liver on glucose metabolism and insulin sensitivity.

The limited research available on the role of additional propionate on DMI of steers fed a high-concentrate diet has shown mixed results with one study indicating no change in DMI (Zhang et al., 2015) and one study showing a dramatic decrease in DMI (33% decrease; Rathert-Williams et al., 2021). The experiments in feedlot cattle have mixed propionate into a portion of the daily ration to dose the animal, and it was concluded that decreased palatability could be a cause of the DMI decrease reported previously (Rathert-Williams et al., 2021). Therefore, it was hypothesized that an increase in ruminal propionate would decrease DMI by decreasing meal size and potentially decreasing meal frequency, along with decreasing insulin sensitivity. The objective of this experiment was to determine if supplying additional propionate directly to the rumen alters DMI, feeding behavior, glucose clearance rate, insulin response, and hepatic gene expression in steers fed a finishing diet.

## Materials and Methods

All animal procedures were approved by the Oklahoma State University Institutional Animal Care and Use Committee (Protocol #19-77).

### Animal management

Six ruminally cannulated Holstein steers (average initial body weight [**BW**] = 418 ± 17.74 [standard deviation] kg) were group housed in feedlot pens (11.27 × 31.85 m with 358.95 m^2^ of the pen covered) at the Oklahoma State University Willard Sparks Beef Research Center (Stillwater, OK) for the duration of the trial with constant access to automatic waterers. Steers were fed the finishing diet (Table 1), ad libitum, for 14 d prior to initiating the experiment. The basal finishing diet was fed once daily in three Insentec Roughage Intake Control system bunks (Hokofarm Group, Marknesse, Netherlands) with adjustments made to insure ad libitum intake. Daily feed intake, number of meals, and meal size were collected by the Insentec system. Meal size was based on weight change of the feed present in each bunk and the timestamp of each visit tracked by each steer’s individual radio-frequency identification tag. Bunk visits within a 10-min period were considered a single meal event (Davis et al., 2014).

**Table 1.** Ingredient and nutrient composition of finishing diet fed throughout the experiment

Ingredient, % of DM	
Rolled corn	62.0
SweetBran^1^	20.0
Prairie hay	8.0
Liquid supplement^2^	5.0
Dry supplement^3^	5.0
Nutrient composition, DM basis	
Dry matter, %	79.51
Crude protein, %	13.50
Neutral detergent fiber, %	23.23
Acid detergent fiber, %	8.60
Ether extract, %	3.91
Ash, %	5.22
NE_m_, Mcal/kg	1.73
NE_g_, Mcal/kg	1.11

^1^SweetBran (Cargill Inc., Dalhart, TX).

^2^Liquid supplement formulated to contain (% DM basis) 45.86% corn steep, 36.17% cane molasses, 6% hydrolyzed vegetable oil, 5.46% 80/20 vegetable oil blend, 5.2% water, 1.23% urea (55% solution), and 0.10 xanthan gum.

^3^Dry supplement formulated to contain (% DM basis) 40.0% ground corn, 29.6% limestone, 20.0% wheat middlings, 7.0% urea, 1.0% salt, 0.53% magnesium oxide, 0.51% zinc sulfate, 0.17% manganese oxide, 0.13% copper sulfate, 0.08% selenium premix (0.6%), 0.0037% cobalt carbonate, 0.32% vitamin A (30,000 IU/g), 0.10% vitamin E (500 IU/g), 0.009% vitamin D (30,000 IU/g), 0.20% tylosin (Tylan-40; Elanco Animal Health, Greenfield, IN), and 0.33% monensin (Rumensin-90; Elanco Animal Health).

### Dietary treatments

Steers were randomly assigned to one of three treatments in a 3 × 6 Latin rectangle: Control (receiving no supplemental propionate); Low Propionate (receiving 100 g/d); or High Propionate (receiving 300 g/d) of Calcium Propionate (Niacet CrystalPro Calcium Propionate; Ingredi, Wilkes-Barre, PA). Treatments were designed to be similar to previous research (Zhang et al., 2015; Rathert-Williams et al., 2021). There were two steers receiving each treatment within each of the 3 periods. Sequence of treatments was balanced for carryover and a 5 d washout period was included between periods where steers did not receive any treatment. Steers were dosed with half of the treatment amount directly through the rumen cannula at 0600 and 1800 hours, daily. A 100 g daily feed sample was collected each day and composited weekly for nutrient analysis. BWs were collected on day 14 to calculate dosing volumes for glucose tolerance tests on day 14 of each period.

### Blood sample collection and analysis

Jugular blood samples were collected on day 7 prior to the morning feeding via jugular venipuncture (9 mL neutral Sarstedt Monovette, Sarstedt AG & Co. KG, Nümbrecht, Germany) with K_2_EDTA added at 1.5 mg/mL, inverted, and immediately placed on ice. Blood samples were centrifuged for 20 min at 3,000 × *g* at 4 °C. Plasma was collected and stored at −20 °C in 2 mL aliquots until further analysis.

An IVGTT was conducted on day 14 of each period following a 12 h fast (Joy et al., 2017; Rathert-Williams et al., 2021). A temporary indwelling jugular catheter (16-gauge × 13 cm; Jorgensen Labs, Loveland, CO) was placed in each steer about 1 h prior to sampling with a 76.2 to 86.4 cm catheter extension set (Oasis, Mettawa, IL). A 2.78 *M* glucose solution was infused at 7.57 mmol/kg BW^0.75^ via the jugular catheter at a continuous rate over 2 min. Blood samples were collected at −10, 0, 5, 10, 15, 20, 25, 30, 45, 60, 90, and 120 min after the glucose infusion (9 mL neutral Sarstedt Monovette, Sarstedt) with K_2_EDTA added at 1.5 mg/mL and immediately placed on ice. Catheters were flushed with 10 mL of heparinized physiological saline (10 IU/mL; ThermoFisher Scientific Chemicals, Inc., Ward Hill, MA) immediately after each blood collection. Blood samples were immediately placed in ice and then samples were centrifuged for 20 min at 3,000 × *g* at 4 °C. Plasma was collected and stored at −20 °C in 2 mL aliquots until further analysis. After completion of each IVGTT, steers were dosed with their respective treatment and monitored for digestive upset after giving access to feed again.

Plasma glucose and ʟ-lactate were analyzed using the YSI Biochemistry Analyzer 2900 (YSI Inc., Yellow Springs, OH). Plasma NEFA were analyzed using a modified protocol of the NEFA-HR (2) kit (Wako Pure Chemical Corporation, Osaka, Japan) based on the acyl-CoA synthetase-acyl-CoA oxidase method. Samples were analyzed in duplicate in 96-well polystyrene plates on a microplate reader (Biotek EPOCH, Biotek Instruments Inc., Winooski, VT) at 550 nm. The intraassay and interassay CV were 6.04% and 4.85%, respectively. Plasma insulin was analyzed using a commercially available porcine insulin radioimmunoassay (**RIA**) kit (Millipore Corporation, Billerica, MA) with insulin from bovine pancreas (Sigma-Aldrich Inc., St. Louis, MO) used to construct a standard curve. Prior to analyzing the samples, linearity and parallelism were verified in four dilutions of a pooled bovine plasma sample that resulted in concentrations ranging from 0.159 to 1.72 µg/L. The RIA kit had a sensitivity of 0.080 ng/mL with a sample size of 100 µL, and 90% specificity to bovine insulin. Samples were prepared for analysis in 12 × 75 mm glass culture tubes and counted in duplicate for 2 min per tube on a 2470 Automatic Gamma Counter (PerkinElmer Inc., Waltham, MA). The intraassay and interassay CV were 2.22% and 2.16%, respectively.

### Rumen fluid collection and analysis

Rumen fluid was serially collected at 0, 2, 4, 6, 8, 10, and 12 h after the morning dosing on day 13 of each period. Samples were collected through a 0.297 mm screen (Rumen Fluid Sampler Tube, Bar Diamond, Parma, ID) attached to 101 cm extension set and a 60 mL syringe. Samples were taken from the cranial and ventral sacs of the rumen. The 0 and 12 h samples were collected prior to each treatment dosing. A total of 50 mL of rumen fluid was collected at each time point. Rumen fluid pH was immediately measured after collection using an Oakton pH 6+ Handheld pH meter (Cole-Parmer, Vernon Hills, IL). ʟ-lactate was analyzed using the YSI Biochemistry Analyzer 2900 (YSI Inc., Yellow Springs, OH). VFA were analyzed using a dimethyl carbonate extraction and gas chromatography with mass spectrometry (Foote, 2022).

### Liver biopsies and gene expression

Liver biopsies were performed on day 15 of each period using a protocol modified from Sexten et al. (2012) and described previously (Rathert-Williams et al., 2021) using an 11-gauge, 15-cm Jamshidi biopsy needle (CareFusion, Vernon Hills, IL). At least 3 samples were collected from the same biopsy site due to the small sampling size of the biopsy needles.

Total RNA of the liver was isolated using the RNeasy Plus Mini Kit and QiaShredder columns (Qiagen, Hilden, Germany) and previously described (Rathert-Williams et al., 2021). About 10 to 30 mg of liver tissue was homogenized in 600 µL of RLT Plus lysis buffer with β-mercaptoethanol using a PowerGen 125 homogenizer (Fisher Scientific, Waltham, MA) for 40 s. The lysate was transferred to a QiaShredder column and centrifuged at 21,100 × *g* for 3 min at room temperature. Following the QiaShredder, the manufacturer’s instructions for the RNeasy Plus Mini kit were followed and the total RNA was eluted in 50 µL of RNase-free water. The total RNA was quantified using a NanoDrop One spectrophotometer (ThermoFisher Scientific, Waltham, MA).

The previously isolated total RNA was used to synthesize cDNA using the iScript cDNA Synthesis Kit per the manufacturer’s protocol (Bio-Rad, Hercules, CA). PrimePCR assays designed by Bio-Rad were used with the SsoAdvanced Universal SYBR Green Supermix to perform quantitative real-time polymerase chain reaction (**RT-qPCR**). Five target genes were selected to analyze, including solute carrier family 16 member 1 (*SLC16A1*), glucose-6-phosphatase (*G6PC*), phosphoenolpyruvate carboxykinase 1 (*PCK1*), phosphoenolpyruvate carboxykinase 2 (*PCK2*), and solute carrier family 2 member 2 (*SLC2A2*) with bovine control gene *G3PDH*. The housekeeping gene *G3PDH* has been previously confirmed to be a suitable housekeeping gene in bovine liver tissue (Lisowski et al., 2008; Lecchi et al., 2012). Table 2 includes the information for the PCR assays used in this experiment including assay ID and reference sequence. Each primer used was tested for efficiency by a serial dilution of a pooled cDNA sample which all showed *R*^2^ > 0.996 and efficiencies between 90% and 100%. Amplicons from the PCR assays were analyzed on an Agilent TapeStation 2200 (Agilent Technologies, Inc; Santa Clara, CA) with a D1000 tape. All amplicons displayed the expected size (Table 2), and a single product was observed (data not shown). All PCR assays did not amplify the negative controls.

**Table 2.** Descriptive information for the PCR assays utilized

Gene symbol	Gene name	Assay ID^1^	Chromosomal location^2^	Transcript^3^	Reference sequence^4^	Amplicon size^5^
*SLC16A1*	Solute carrier family 16 member 1	qBtaCID0003185	3: 30382285–30411742	ENSBTAT00000020102	NM_001037319	119
*G6PC*	Glucose-6-phosphatase	qBtaCID0004949	19: 42949691–42960484	ENSBTAT00000013436	NM_001076124	86
*PCK1*	Phosphoenolpyruvate carboxykinase 1	qBtaCED0009985	13: 58609003–58615151	ENSBTAT00000002520	NM_174737	101
*PCK2*	Phosphoenolpyruvate carboxykinase 2	qBtaCED0011018	10: 21036261–21044548	ENSBTAT00000015834	NM_001205594	116
*SLC2A2*	Solute carrier family 2 member 2	qBtaCID0012841	1: 96452924–96783056	ENSBTAT00000055725	NM_001103222	113
*G3PDH*	Glucose-3-phosphate dehydrogenase	qBtaCID0013312	5: 103870384–103874667	ENSBTAT00000037753	NM_001244135.2	92

All PCR assays are intron spanning and single products were verified by melt curves and on an Agilent TapeStation 2200 (Agilent Technologies, Inc; Santa Clara, CA) with a D1000 tape.

^1^Unique assay identifier for Bio-Rad Prime PCR Assay (Bio-Rad, Hercules, CA).

^2^Specific chromosomal location of the targeted transcript on the ARS-UCD1.2 bovine genome assembly.

^3^Targeted transcript information identifier from Ensembl (ensembl.org).

^4^National Center for Biotechnology Information (NCBI) reference sequence used for primer design by Bio-Rad.

^5^Expected size (base pairs) of amplicon as reported by the manufacturer. Size of amplicons were verified using an Agilent TapeStation 2200 (Agilent Technologies, Inc; Santa Clara, CA) with a D1000 tape.

RT-qPCR was performed in triplicate for each cDNA sample using 10 µL of SsoAdvanced Universal SYBR Green Supermix, 1 µL of each PrimePCR assay, 7 µL of nuclease-free water, and 2 µL of diluted cDNA sample template. The reaction was performed using a Bio-Rad CFX96 real-time PCR detection instrument with the following protocol: 95 °C for 30 s, 40 cycles of 95 °C for 10 s, and 60 °C for 30 s, and a final melting curve from 65 to 95 °C for 5 s. The threshold cycle (C_p_) for each sample was determined and used to calculate 2^−ΔΔCt^ along with the control primer and pooled cDNA sample.

### Statistical analysis

Data from the IVGTT were analyzed using GraphPad Prism 8.4.3 (GraphPad Software, San Diego, CA) to determine the area under the curve (**AUC**) for glucose and insulin. This data was also modeled as an exponential one-phase decay to calculate glucose and insulin peak, rate, and plateau (GraphPad Prism). Additionally, data from the IVGTT were used to calculate surrogate insulin sensitivity indices including the homeostatic model assessment for insulin resistance (**HOMA-IR**) and quantitative insulin sensitivity check index (**QUICKI**) as described previously (Rathert-Williams et al., 2021). All other data were analyzed using the MIXED procedure of SAS 9.4 (SAS Institute Inc., Cary, NC) with period, treatment, and day as fixed effects and steer as a random effect. Day was considered a repeated measure for DMI, number of meals, and meal size. Hour was considered a repeated measure for rumen fluid lactate, pH, and VFA. Minute was considered a repeated measure for plasma insulin and glucose concentrations. Covariance structure for repeated measures was chosen from autoregressive, compound symmetry, unstructured, and variance components based on the lowest Akaike Information Criterion. Autoregressive covariance structure was used for all analyses. Residual plots were analyzed for normality and variables found to display a non-normal distribution of residuals were log_10_ transformed for analysis. Means were separated using the LS-Means statement with a Tukey–Kramer post hoc adjustment. Means presented are least-square means ± standard error of the mean (**SEM**). Log-transformed means are presented as both the log-transformed means (± SEM) and the back-transformed means. Means were considered significantly different if *P* ≤ 0.05 and were considered tendencies if 0.05 < *P* ≤ 0.10.

## Results

**Figure 1.**
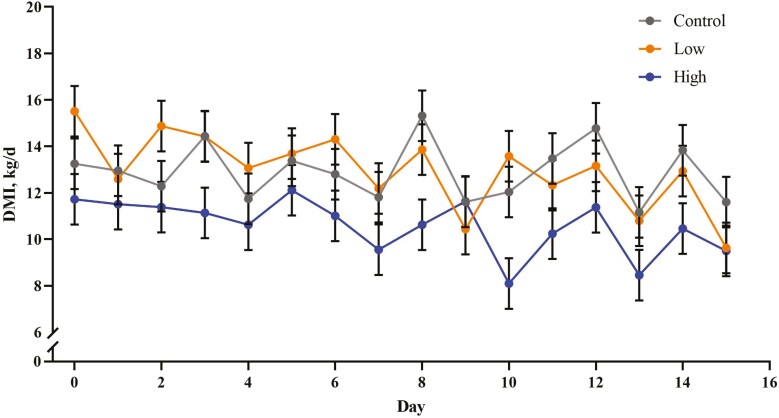
Treatment × time interaction for DMI (kg/d; *P* = 0.65) in steers receiving Control (0 g/d, ●), Low (100 g/d, ●), or High (300 g/d, ●) calcium propionate treatments. DMI was evaluated in three 15-d experimental periods. Treatments were arranged in a 3 × 6 Latin rectangle.

DMI was decreased in the High treatment (Table 3; *P* < 0.001) while the Control and Low treatments were similar. The decrease in DMI in High propionate-treated steers was due to both a decrease in meal size (*P* = 0.049) and the number of meals per day (*P* = 0.046) compared to the Low and Control treatment. The treatment × day interaction for DMI is shown in Figure 1 for comparison to previous experiments. Plasma insulin measured pre-feeding on day 7 of each period was decreased in the High treatment compared to the Control (Table 4; *P* = 0.008) with the Low treatment intermediate to the other treatments. There was no effect of propionate treatment on pre-feed day 7 plasma glucose concentration (*P* = 0.23) or lactate concentration (*P* = 0.47), but NEFA ­concentrations were increased in the High treatment (*P* = 0.044) compared to the Control.

**Table 3.** Effect of calcium propionate dosed at 0 g/d (Control), 100 g/d (Low), or 300 g/d (High) on BW, DMI, and feeding behavior in 15 d experimental periods

	Treatments^1^				*P*-values		
Variable	Control	Low	High	SEM^2^	Treatment	Day	Treatment × day
BW, kg	475.2	477.5	469.7	7.15	0.65	–	–
DMI, kg/d	12.9^a^	13.0^a^	10.6^b^	0.30	<0.001	<0.001	0.65
DMI, %/BW	2.87^a^	2.81^a^	2.32^b^	0.081	<0.001	<0.001	0.73
Meal size, kg	1.60^ab^	1.62^a^	1.45^b^	0.120	0.049	0.015	0.78
Meal size, %/BW	0.36^a^	0.35^ab^	0.32^b^	0.029	0.024	0.012	0.81
Meal frequency, n/d	8.40^a^	8.40^a^	7.75^b^	0.511	0.046	<0.001	0.91

Treatments were arranged in a 3 × 6 Latin rectangle.

^1^Treatments included: Control, 0 g/d calcium propionate; Low, 100 g/d calcium propionate; High, 300 g/d calcium propionate.

^2^Standard error of the mean (*n* = 6).

^a,b^Values within row with differing superscripts differ (*P* < 0.05).

**Table 4.** Effect of calcium propionate dosed at 0 g/d (Control), 100 g/d (Low), or 300 g/d (High) on day 7 pre-feeding plasma metabolites and insulin concentrations of steers fed a finishing diet

Variable	Treatments^1^			SEM^2^	*P*-value
	Control	Low	High		
Insulin, ng/mL	1.252^a^	0.937^ab^	0.737^b^	0.1030	0.008
Glucose, mg/dL	79.1	76.2	76.7	1.61	0.23
Lactate, mg/dL	10.17	9.27	9.92	0.688	0.47
NEFA, µEq/L	76.8^b^	85.7^ab^	102.4^a^	6.40	0.044

Treatments were arranged in a 3 × 6 Latin rectangle.

^1^Treatments included: Control, 0 g/d calcium propionate; Low, 100 g/d calcium propionate; High, 300 g/d calcium propionate.

^2^Standard error of the mean (*n* = 6).

**Figure 2.**
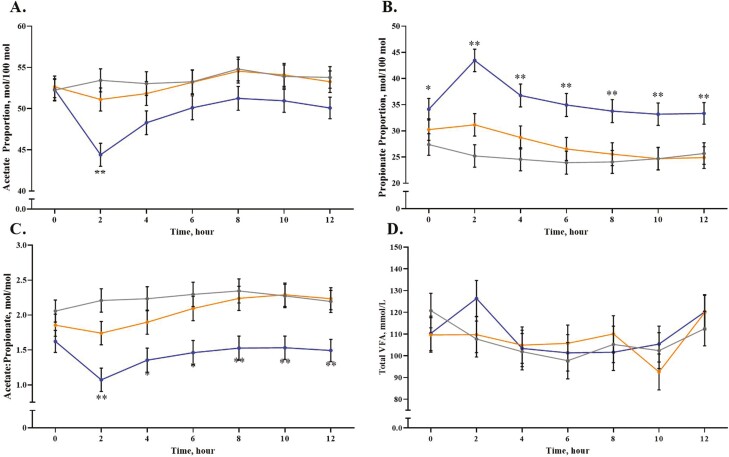
Treatment × time interaction for the proportion of acetate (A; *P* < 0.001), propionate (B; *P* < 0.001), the ratio of acetate:propionate (C; *P* = 0.005), and total VFA concentrations (D; *P* = 0.14) in steers receiving Control (0 g/d, ●), Low (100 g/d, ●), or High (300 g/d, ●) calcium propionate treatments. Treatments were arranged in a 3 × 6 Latin rectangle. Rumen fluid samples were collected on day 13 of each experimental period.

There was no treatment × time interaction for rumen lactate concentration (*P* = 0.84; Table 5), pH (*P* = 0.74), or total VFA concentrations (*P* = 0.14). Rumen fluid lactate was decreased in the High propionate treatment (*P* = 0.015) compared to the Low treatment and the Control was intermediate and did not differ from either treatment. There was a tendency (*P* = 0.06) for a time effect on lactate concentrations due to concentrations increasing after the 0 h sampling (data not shown). Treatment did not affect (*P* = 0.69) rumen fluid pH but there was an effect of time (*P* < 0.001), where pH increased after 0 h was consistent throughout sampling and decreased slightly at 12 h. Total VFA concentrations in the rumen fluid were not affected by treatment (*P* = 0.88). The effect of time (*P* < 0.001) was due to a difference in concentrations at 10 h (100.1 mM) versus 12 h (117.5 mM), with all other timepoints displaying an intermediate value and not differing from the other timepoints (data not shown). While there was no effect of treatment on total VFA concentrations, there was a treatment × time interaction for acetate (*P* < 0.001; Figure 2), propionate (*P* < 0.001), and the ratio of acetate:propionate (*P* = 0.005). Acetate proportions were decreased in the High propionate treatment at 2 h compared to the Low treatment and Control (*P* = 0.005) but did not differ at the other time points (*P* > 0.05). Propionate proportions were increased in the High treatment compared to the Control (*P* < 0.001) at 0 h but not the Low treatment. At all other timepoints, the High treatment displayed a greater propionate proportion (*P* < 0.001) than both the Low treatment and control. The acetate:propionate ratio was decreased in the High treatment compared to both other groups at 2, 8, 10, and 12 h after dosing with the treatment and only differed from the Control at 4 and 6 h. Butyrate proportions were decreased in the High treatment compared to both other treatments (*P* < 0.001). Valerate proportions were greatest in the High treatment (*P* = 0.001) compared to the Low and Control. There were minimal effects on the proportions of branch-chain VFA, with isobutyrate displaying a tendency (*P* = 0.07) to decrease in the High treatment and no effect of treatment on isovalerate proportions (*P* = 0.12).

**Table 5.** Effect of calcium propionate dosed at 0 g/d (Control), 100 g/d (Low), or 300 g/d (High) on rumen fluid lactate, pH, and VFA

	Treatments^1^				*P*-values		
Variable	Control	Low	High	SEM^2^	Treatment	Time	Treatment × time
Log lactate	−0.26^ab^	−0.16^a^	−0.52^b^	0.109	0.015	0.06	0.84
ʟ-Lactate, mg/dL	0.55	0.69	0.30	–	–	–	–
pH	6.26	6.37	6.33	0.120	0.69	<0.001	0.74
Total VFA, mM^3^	106.9	107.5	109.8	5.82	0.88	<0.001	0.14
VFA proportions, mol/100 mol							
Acetate^3^	53.5^a^	53.0^a^	49.6^b^	1.12	0.002	<0.001	<0.001
Propionate^3^	25.1^b^	27.4^b^	35.7^a^	1.92	<0.001	<0.001	<0.001
Isobutyrate	0.82	0.78	0.67	0.057	0.07	0.018	0.12
Butyrate	17.9^a^	16.2^a^	11.0^b^	1.13	<0.001	0.005	0.41
Isovalerate	0.78	0.73	0.64	0.061	0.12	0.001	0.26
Valerate	1.89^b^	1.79^b^	2.34^a^	0.123	0.001	<0.001	0.63
Acetate:Propionate, mol/mol^3^	2.23	2.05	1.44	0.141	<0.001	0.002	0.005

Treatments were arranged in a 3 × 6 Latin rectangle.

^1^Treatments included: Control, 0 g/d calcium propionate; Low, 100 g/d calcium propionate; High, 300 g/d calcium propionate.

^2^Standard error of the mean (*n* = 6).

^3^Treatment × time interactions are presented in Figure 2.

^a,b^Values within row with differing superscripts differ (*P* < 0.05).

There was no effect of treatment on fasting plasma concentrations of glucose, lactate, or insulin (Table 6; *P* ≥ 0.44) as measured before the glucose infusion. Additionally, there was no treatment effect on glucose peak, plateau, or clearance rate, and plasma insulin or glucose AUC (*P* ≥ 0.50). A tendency (*P* = 0.09) was observed for the insulin time to peak due to the Control have a quicker time to peak than the High and Low treatments. There was no treatment effect (*P* ≥ 0.34) on HOMA-IR or QUICKI surrogate insulin indices. Propionate treatment did not affect liver gene expression (Table 7; *P* ≥ 0.57).

**Table 6.** Effect of calcium propionate dosed in the rumen at 0 g/d (Control), 100 g/d (Low), or 300 g/d (High) on fasting plasma metabolites and insulin concentrations and parameters from the IVGTT of steers fed a finishing diet

	Treatments^1^				
Variable	Control	Low	High	SEM^2^	*P*-value
Fasting glucose, mg/dL	80.5	79.8	79.7	1.76	0.93
Fasting log lactate	0.947	0.991	0.924	0.0414	0.45
Lactate, mg/dL	9.27	8.52	9.85	–	–
Fasting log insulin	−0.203	−0.277	−0.360	0.0821	0.44
Insulin, ng/mL	0.627	0.528	0.437	–	–
Insulin AUC	266.1	258.8	223.7	29.13	0.50
Insulin peak, ng/mL	6.34	5.88	5.22	0.915	0.61
Insulin time to peak, min	9.17	15.83	13.33	1.839	0.09
Glucose AUC	15,703.0	14,984.0	15,572.0	486.3	0.56
Glucose peak, mg/dL	466.2	429.3	510.9	70.54	0.72
Glucose plateau, mg/dL	95.4	89.2	90.2	5.96	0.72
Glucose clearance rate	0.164	0.147	0.180	0.0383	0.83
HOMA-IR^3^	81.5	63.2	51.7	13.48	0.34
QUICKI^4^	0.325	0.332	0.341	0.0098	0.53

Treatments were arranged in a 3 × 6 Latin rectangle. The glucose tolerance test was administered on day 14 of each 15 d experimental period.

^1^Treatments included: Control, 0 g/d calcium propionate; Low, 100 g/d calcium propionate; High, 300 g/d calcium propionate.

^2^Standard error of the mean (*n* = 6).

^3^HOMA-IR, homeostatic model assessment for insulin resistance.

^4^QUICKI, quantitative insulin sensitivity check index.

**Table 7.** Effect of calcium propionate dosed at 0 g/d (Control), 100 g/d (Low), or 300 g/d (High) on the hepatic expression of genes required for gluconeogenesis in steers fed a finishing diet

	Treatments^1^				
Gene	Control	Low	High	SEM^2^	*P*-value
*SLC16A1*	0.038	0.084	0.051	0.0741	0.87
*G6PC*	−0.019	−0.029	0.008	0.0668	0.92
*PCK1*	−0.139	−0.067	−0.019	0.0849	0.63
*PCK2*	−0.101	−0.049	−0.081	0.0781	0.89
*SLC2A2*	−0.357	−0.298	−0.267	0.0573	0.57

Liver samples were collected by biopsy on day 15 of each experimental period. Treatments were arranged in a 3 × 6 Latin rectangle.

^1^Treatments included: Control, 0 g/d calcium propionate; Low, 100 g/d calcium propionate; High, 300 g/d calcium propionate.

^2^Standard error of the mean (*n* = 6).

## Discussion

In contrast to a previous experiment (Rathert-Williams, 2021), DMI was only reduced with the addition of 300 g/d, where the previous study reported a decrease in the 100 g/d treatment and a further decrease in the 300 g/d treatment. The effect of propionate on DMI and feeding behavior in the literature is inconsistent due to the variation in dose amount, mode of administering the dose, and the production period targeted. In contrast to the current study, DMI was not affected by increasing propionate supplementation in transition and early lactation dairy cows fed 228 or 300 g/d (DeFrain et al., 2005; Liu et al., 2010) or in finishing cattle dosed with 200 g Ca propionate per day (Zhang et al., 2015). However, an increase in DMI was reported when early lactation cows were supplied 125 g/d calcium propionate in a pelleted form (McNamara and Valdez, 2005). When compared to an intraruminal acetate infusion, propionate decreased DMI in early lactation dairy cows by 20% over an 18 h infusion period (Stocks and Allen, 2012). Sheperd and Combs (1998) reported a similar effect in lactating cows with a continuous ruminally infused propionate dose on a high forage diet. Oba and Allen (2003b) attribute this depressed DMI to the hypophagic effects of propionate, altering satiety, and hunger signals. Some of the differences in results from separate experiments could be due to the mode of administering the treatments (i.e., continuous infusion, bolus dosing, or feeding). In theory, the administration of a bolus dose twice daily could result in transient large increases in propionate concentrations and supply, but data here indicate that ruminal propionate proportions stay consistent throughout the day with a bolus dose. The literature is fairly inconsistent with regard to infusion duration, concentration of infusate, and other factors that make comparing results tenuous.

Even though propionate was administered directly into the rumen, it is unlikely that propionate supply to the liver was increased. Using an average propionate production rate (167 g propionate per kg DMI; Bauman et al., 1971; Yost et al., 1977) the expected propionate production rate from fermentation of the diet for the observed DMI from each treatment were 2.15, 2.17, and 1.77 kg/d for the Control, Low, and High treatments, respectively. Therefore, propionate production in the High treatment is 384 g/d lower than the Control. The reduction of 384 g/d is 84 g/d greater than the amount supplemented. The calculation of propionate production based on DMI is reliant on the assumption that fermentation is not disrupted by adding propionate to the rumen. The lack of an effect on rumen fluid pH and total VFA concentrations indicates that supplying additional propionate into the rumen does not disrupt fermentation and suggests that the assumptions of propionate production could be justified. The slight decrease in rumen lactate concentrations could indicate that rumen fermentation patterns are shifted in response to the introduction of exogenous propionate. The difference in lactate concentrations was only observed between the Low and High treatments, which would indicate that adding 300 g/d of propionate did not alter lactate production or utilization, further supporting the assumption that the literature values for propionate production are reliable. The influence of adding fermentation end products on rumen dynamics and VFA production should be evaluated as a potential regulation point of DMI, digestion, and nutrient utilization efficiency.

The satiating effect of propionate has been speculated to be mediated through hepatic oxidation of acetyl-CoA through the tricarboxylic acid cycle (Allen et al., 2009). The oxidation of acetyl-CoA is thought to decrease the rate of firing of the hepatic vagus nerve signaling satiety during a meal (Anil and Forbes, 1988). The observation of a decreased meal size in the current study has been previously reported in lactating dairy cows when propionate was infused intraruminally for 12 h (Oba and Allen, 2003b). However, a conflicting report indicated no effect of ruminal propionate infusion on meal size over 18 h (Stocks and Allen, 2012). The current study is interesting in that the decrease in meal size and frequency was observed over several days with twice daily dosing. It is yet to be determined if the effect of additional propionate supplied to the rumen of cattle consuming a high-concentrate finishing diet on meal size and overall DMI is repeatable, but the differences between the current study and previous reports could be due to diet differences and metabolic demands of production (i.e., lactation vs. growth). Additionally, given the discussion above, it is unclear and unlikely that the treatments in the current experiment and previous experiment (Rathert-Williams et al., 2021) actually increase propionate supply to the liver of cattle and it is likely that other mechanisms are responsible for depressions in DMI.

A possible mechanism for the effect of increasing ruminal propionate supply on decreasing feed intake is the role of rumen osmolality in regulating feed intake. Previous studies have shown that increasing osmolality in the rumen decreases appetite (Carter and Grovum, 1990). The same effect has been shown with increase osmolality using acetate, propionate, and butyrate (Ternouth and Beattie, 1971). It is possible that a portion of the decrease in feed intake observed with increasing rumen propionate is due to an increase in osmolality of the rumen environment. However, Carter and Grovum (1990) indicate that the effect of hyperosmolality on appetite is relatively short-lived (approximately 1 h). Given the short-lived nature of the effect, as well as the lack of an increase in total VFA concentrations, it is unclear what role rumen osmolality played in the observed effects on DMI. The role of osmolality in regulating DMI should be evaluated in future experiments.

One speculation of increasing propionate supply is that it could increase gluconeogenesis by the liver in response to the increased substrate. The increase in glucose production could in turn have effects on insulin sensitivity and whole-body glucose metabolism. In mature goats, infusing propionate into the rumen resulted in an increase in plasma insulin (Stern et al., 1970). In the previous study using steers consuming a similar diet to the current experiment, increasing propionate through top-dressing the diet, increased the amount of insulin released in response to an IVGTT (Rathert-Williams et al., 2021). In contrast to the previous experiment, additional propionate had no major effects on glucose metabolism or insulin sensitivity. The only minor effect observed in the current study on insulin signaling was that day 7 pre-feeding insulin concentrations were decreased in the High treatment and there was a slight increase in the insulin time to peak. The decrease in pre-feeding insulin was not observed during fasting conditions prior to the IVGTT and could be in response to the depressed feed intake. Additionally, as discussed above, it does not appear that the treatments used in this experiment increased propionate supply to the animal, which could explain the lack of an effect on insulin activity and glucose metabolism.

Similar to Rathert-Williams et al. (2021), an increase in pre-feeding NEFA concentrations was observed in this experiment. This increase in circulating NEFA is consistent with the decrease in insulin and DMI, as cattle were likely mobilizing body fat under the hypoinsulemic conditions. Additionally, the increased mobilization of NEFA would provide more oxidative fuels to the liver and in theory contribute to the depressed DMI (Allen et al., 2009).

Expression of *SLC16A1* and *SLC2A2* were previously shown to be increased with propionate fed to steers (Rathert-Williams et al., 2021). It was speculated that increasing propionate could lead to an increase in the protein that mediates propionate uptake by the liver (monocarboxylate transporter 1 [MCT1] encoded by *SLC16A1*) and an increase in glucose exported by the liver via glucose transporter 2 (GLUT2 encoded by *SLC2A2*). However, it is not possible to know for sure as gene expression studies do not provide information on protein expression or function and transporter capacity. The lack of a response in gene expression in the current study could indicate that propionate concentrations in the portal vein did not increase enough to evoke an increase in the expression of metabolic pathways that metabolize propionate or that the pathways possess enough capacity to handle the increased flux of propionate. It should also be noted that the steers in the previous study (Rathert-Williams et al., 2021) were on the propionate treatments for 33 d compared to 15 d in the current experiment which could contribute to some of the differences between experiments. These gene expression data from the current experiment would support the HOT that increasing propionate results in increased hepatic oxidation thereby decreasing DMI, yet previous research (Rathert-Williams et al., 2021) indicates that some of the propionate may be shunted away from oxidation and through gluconeogenesis.

## Conclusion

This experiment suggests that supplying ruminal propionate to steers fed a finishing ration decreases DMI by altering feeding behavior even in the absence of potential palatability issues. The decrease in both meal size and meal frequency supports previous research that rapid uptake of propionate to the liver causes satiety and potentially the excess propionate available for oxidation can increase meal intervals. However, the depression of DMI likely results in a decreased total VFA production from fermentation that negates the supply of propionate from the exogenous treatment. It is therefore unlikely that the depression in DMI is due to an increased supply of propionate to the liver. It is possible that additional propionate in the rumen alters rumen dynamics and fermentation that could contribute to decreased DMI. Pulse dosing nutrient such as propionate may affect post-ruminal flows and affect DMI regulation. It appears that rumen fermentation end products are important in the regulation of DMI, but the mechanisms are yet to be determined.

## Abbreviations

AIC: Akaike Information Criterion
AUC: area under the curve
BW: body weight
DMI: dry matter intake
G6PC: glucose-6-phosphatase
GLUT2: glucose transporter 2
HEC: hyperinsulinemic-euglycemic clamp
HOMA-IR: homeostatic model assessment for insulin resistance
HOT: hepatic oxidation theory
IVGTT: intravenous glucose tolerance test
MCT1: monocarboxylate transporter 1
ME: metabolizable energy
NEFA: non-esterified fatty acids
PCK1: phosphoenolpyruvate carboxykinase 1
PCK2: phosphoenolpyruvate carboxykinase 2
PEPCK: phosphoenolpyruvate carboxykinase
QUICKI: quantitative insulin sensitivity check index
RFID: radio-frequency identification
RIA: radioimmunoassay
RT-qPCR: quantitative reverse transcription polymerase chain reaction
SLC16A1: solute carrier family 16 member 1
SLC2A2: solute carrier family 2 member 2
TCA cycle: tricarboxylic acid cycle
VFA: volatile fatty acids

## References

[CIT0001] Allen, M. S., B. J. Bradford, and M. Oba. 2009. Board-invited review: the hepatic oxidation theory of the control of feed intake and its application to ruminants. J. Anim. Sci. 87(10):3317–3334. doi:10.2527/jas.2009-1779 19648500

[CIT0002] Allen, M. S. 2014. Drives and limits to feed intake in ruminants. Anim. Prod. Sci. 54:1513–1524. doi:10.1071/an14478

[CIT0003] Anil, M. H., and J. M. Forbes. 1988. The roles of hepatic nerves in the reduction of food intake as a consequence of intraportal sodium propionate administration in sheep. Q. J. Exp. Physiol. 73:539–546. doi:10.1113/expphysiol.1988.sp003174 3174914

[CIT0004] Aschenbach, J. R., N. B. Kristensen, S. S. Donkin, H. M. Hammon, and G. B. Penner. 2010. Gluconeogenesis in dairy cows: the secret of making sweet milk from sour dough. IUBMB Life 62:869–877. doi:10.1002/iub.400 21171012

[CIT0005] Bauman, D. E., C. L. Davis, and H. F. Bucholtz. 1971. Propionate production in the rumen of cows fed either a control or high-grain, low-fiber diet. J. Dairy Sci. 54:1282–1287. doi:10.3168/jds.S0022-0302(71)86021-6 4937668

[CIT0006] Bell, A. W. 1995. Regulation of organic nutrient metabolism during transition from late pregnancy to early lactation. J. Anim. Sci. 73:2804–2819. doi:10.2527/1995.7392804x 8582872

[CIT0007] Carter, R. R., and L. W. Grovum. 1990. Factors affecting the voluntary intake of food by sheep: 5. The inhibitory effect of hypertonicity in the rumen. Br. J. Nutr. 64:285–299. doi:10.1079/BJN19900029 2400766

[CIT0008] Davis, M. P., H. C. Freetly, L. A. Kuehn, and J. E. Wells. 2014. Influence of dry matter intake, dry matter digestibility, and feeding behavior on body weight gain of beef steers. J. Anim. Sci. 92:3018–3025. doi:10.2527/jas.2013-6518 24802034

[CIT0009] DeFrain, J. M., A. R. Hippen, K. F. Kalscheur, and R. S. Patton. 2005. Effects of feeding propionate and calcium salts of long-chain fatty acids on transition dairy cow performance. J. Dairy Sci. 88:983–993. doi:10.3168/jds.S0022-0302(05)72766-1 15738233

[CIT0010] DiCostanzo, A., J. E. Williams, and D. H. Keisler. 1999. Effects of short- or long-term infusions of acetate or propionate on luteinizing hormone, insulin, and metabolite concentrations in beef heifers. J. Anim. Sci. 77:3050–3056. doi:10.2527/1999.77113050x 10568477

[CIT0011] Ferreira, L. S., and C. M. M. Bittar. 2011. Performance and plasma metabolites of dairy calves fed starter containing sodium butyrate, calcium propionate or sodium monensin. Animal. 5:239–245. doi:10.1017/S1751731110001965 22440769

[CIT0012] Foote, A. P. 2022. Technical note: Analysis of volatile fatty acids in rumen fluid by gas chromatography mass spectrometry using a dimethyl carbonate extraction. J. Anim. Sci. doi:10.1093/jas/skac207 PMC9412176 35660871

[CIT0013] Joy, F., J. J. McKinnon, S. Hendrick, P. Górka, and G. B. Penner. 2017. Effect of dietary energy substrate and days on feed on apparent total tract digestibility, ruminal short-chain fatty acid absorption, acetate and glucose clearance, and insulin responsiveness in finishing feedlot cattle1. J. Anim. Sci. 95:5606–5616. doi:10.2527/jas2017.1817 29293742 PMC6292318

[CIT0014] Lalman, D. L., M. K. Petersen, R. P. Ansotegui, M. W. Tess, C. K. Clark, and J. S. Wiley. 1993. The effects of ruminally undegradable protein, propionic acid, and monensin on puberty and pregnancy in beef heifers. J. Anim. Sci. 71: 2843–2852. doi:10.2527/1993.71112843x 8270506

[CIT0015] Lecchi, C., F. Dilda, P. Sartorelli, and F. Ceciliani. 2012. Widespread expression of SAA and hp RNA in bovine tissues after evaluation of suitable reference genes. Vet. Immunol. Immunopathol. 145:556–562. doi:10.1016/j.vetimm.2011.12.017 22230385

[CIT0016] Lisowski, P., M. Pierzchała, J. Gościk, C. S. Pareek, and L. Zwierzchowski. 2008. Evaluation of reference genes for studies of gene expression in the bovine liver, kidney, pituitary, and thyroid. J. Appl. Genet. 49:367–372. doi:10.1007/BF03195635 19029684

[CIT0017] Liu, Q., C. Wang, W. Z. Yang, G. Guo, X. M. Yang, D. C. He, K. H. Dong, and Y. X. Huang. 2010. Effects of calcium propionate supplementation on lactation performance, energy balance and blood metabolites in early lactation dairy cows. J. Anim. Physiol. Anim. Nutr. (Berl) 94:605–614. doi:10.1111/j.1439-0396.2009.00945.x 19906132

[CIT0018] McNamara, J. P., and F. Valdez. 2005. Adipose tissue metabolism and production responses to calcium propionate and chromium propionate. J. Dairy Sci. 88:2498–2507. doi:10.3168/jds.S0022-0302(05)72927-1 15956312

[CIT0019] Oba, M., and M. S. Allen. 2003a. Effects of intraruminal infusion of sodium, potassium, and ammonium on hypophagia from propionate in lactating dairy cows. J. Dairy Sci. 86:1398–1404. doi:10.3168/jds.S0022-0302(03)73723-0 12741564

[CIT0020] Oba, M., and M. S. Allen. 2003b. Intraruminal infusion of propionate alters feeding behavior and decreases energy intake of lactating dairy cows. J. Nutr. 133:1094–1099. doi:10.1093/jn/133.4.1094 12672925

[CIT0021] Owens, F. N., and A. L. Goetsch. 1993. Ruminal fermentation. In: D. C. Church, editor. The ruminant animal: digestive physiology and nutrition. Long Grove (IL): Waveland Press, Inc; p 145–171.

[CIT0022] Rathert-Williams, A. R., C. M. Salisbury, A. K. Lindholm-Perry, A. Pezeshki, D. L. Lalman, and A. P. Foote. 2021. Effects of increasing calcium propionate in a finishing diet on dry matter intake and glucose metabolism in steers. J. Anim. Sci. 99. doi:10.1093/jas/skab314 PMC8645227 34718608

[CIT0023] Sexten, A. K., C. R. Krehbiel, J. W. Dillwith, R. D. Madden, C. P. McMurphy, D. L. Lalman, and R. G. Mateescu. 2012. Effect of muscle type, sire breed, and time of weaning on fatty acid composition of finishing steers. J. Anim. Sci. 90:616–625. doi:10.2527/jas.2011-4218 21965455

[CIT0024] Sheperd, A. C., and D. K. Combs. 1998. Long-term effects of acetate and propionate on voluntary feed intake by midlactation cows. J. Dairy Sci. 81:2240–2250. doi:10.3168/jds.S0022-0302(98)75803-5 9749390

[CIT0025] Stern, J. S., C. A. Baile, and J. Mayer. 1970. Are propionate and butyrate physiological regulators of plasma insulin in ruminants? American Journal of Physiology. 219(1):84–91. doi:10.1152/ajplegacy.1970.219.1.84 5424862

[CIT0026] Stocks, S. E., and M. S. Allen. 2012. Hypophagic effects of propionate increase with elevated hepatic acetyl coenzyme a concentration for cows in the early postpartum period. J. Dairy Sci. 95:3259–3268. doi:10.3168/jds.2011-4991 22612960

[CIT0027] Ternouth, J. H., and A. W. Beattie. 1971. Studies of the food intake of sheep at a single meal. Br. J. Nutr. 25:153–164. doi:10.1079/bjn19710073 5539289

[CIT0028] Wang, L., G. Zhang, Y. Li, and Y. Zhang. 2020. Effects of high forage/concentrate diet on volatile fatty acid production and the microorganisms involved in VFA production in cow rumen. Animals. 10:223. doi:10.3390/ani10020223 32019152 PMC7070707

[CIT0029] Yost, W. M., J. W. Young, S. P. Schmidt, and A. D. McGilliard. 1977. Gluconeogenesis in ruminants: propionic acid production from a high-grain diet fed to cattle. J. Nutr. 107:2036–2043. doi:10.1093/jn/107.11.2036 908961

[CIT0030] Zhang, X. Z., Q. X. Meng, L. Lu, Z. L. Cui, and L. P. Ren. 2015. The effect of calcium propionate supplementation on performance, meat quality, and mRNA expression of finishing steers fed a high-concentrate diet. J. Anim. Feed Sci. 24:100–106. doi:10.22358/jafs/65634/2015

